# Species C Rotaviruses in Children with Diarrhea in India, 2010–2013: A Potentially Neglected Cause of Acute Gastroenteritis

**DOI:** 10.3390/pathogens7010023

**Published:** 2018-02-17

**Authors:** Sudipta Bhat, Jobin Jose Kattoor, Yashpal Singh Malik, Shubhankar Sircar, Pallavi Deol, Vinita Rawat, Ritu Rakholia, Souvik Ghosh, Anastasia N. Vlasova, Touil Nadia, Kuldeep Dhama, Nobumichi Kobayashi

**Affiliations:** 1 Division of Biological Standardization, ICAR-Indian Veterinary Research Institute, Izatnagar, Bareilly 243122, India; sudiptabhat1991@gmail.com (S.B.); jobinjkattoor@gmail.com (J.J.K.); shubhankar.sircar@gmail.com (S.S.); pallavi.deol@gmail.com (P.D.); 2 Department of Microbiology, Government Medical College, Haldwani, Nainital, Uttarakhand 263 139, India; drvinitarawat@gmail.com; 3 Department of Pediatrics, Government Medical College, Haldwani, Nainital, Uttarakhand 263 139, India; lalitriturakholia@rediffmail.com; 4 Department of Biomedical Sciences, One Health Center for Zoonoses and Tropical Veterinary Medicine, Ross University School of Veterinary Medicine, P.O. Box 334, Basseterre, St. Kitts, West Indies; souvikrota@gmail.com; 5 Food Animal Health Research Program, CFAES, Ohio Agricultural Research and Development Center, Department of Veterinary Preventive Medicine, The Ohio State University, Wooster, OH 44691, USA; vlasova.1@osu.edu; 6 Laboratoire de Biosécurité et de Recherche, Hôpital Militaire d'Instruction Med V de Rabat; 110 000 Morocco; ntouil2003@gmail.com; 7 Division of Pathology, ICAR-Indian Veterinary Research Institute, Izatnagar, Bareilly 243 122, India; kdhama@rediffmail.com; 8 Sapporo Medical University School of Medicine, Chuo-Ku, Sapporo 060-8556, Japan; koba103161@yahoo.co.jp

**Keywords:** rotavirus C, acute gastroenteritis, sequence analysis, phylogenetic analysis, VP6, VP4, NSP4 genes, India

## Abstract

All over the world, children and adults are severely affected by acute gastroenteritis, caused by one of the emerging enteric pathogens, rotavirus C (RVC). At present, no extensive surveillance program is running for RVC in India, and its prevalence is largely unknown except cases of local outbreaks. Here, we intended to detect the presence of RVC in diarrheic children visiting or admitted to hospitals in Haldwani (state of Uttarakhand, India), a city located in the foothills of the Himalayas. During 2010–2013, we screened 119 samples for RVC by an RVC VP6 gene-specific RT-PCR. Of these, 38 (31.93%) were found positive, which is higher than the incidence rates reported so far from India. The phylogenetic analysis of the derived nucleotide sequences from one of the human RVC (HuRVC) isolates, designated as HuRVC/H28/2013/India, showed that the study isolate belongs to genotype I2, P2 and E2 for RVC structural genes 6 and 4 (VP6, and VP4) and non-structural gene 4 (NSP4), respectively. Furthermore, the VP6 gene of HuRVC/H28/2013/India shows the highest similarity to a recently-reported human-like porcine RVC (PoRVC/ASM140/2013/India, KT932963) from India suggesting zoonotic transmission. We also report a full-length NSP4 gene sequence of human RVC from India. Under the One-health platforms there is a need to launch combined human and animal RVC surveillance programs for a better understanding of the epidemiology of RVC infections and for implementing control strategies.

## 1. Introduction

Acute gastroenteritis (AGE) is one of the prevailing cause leading to high mortality and morbidity worldwide among humans and animals. It is estimated that every year, between three and five billion cases and 1.5–2.5 million deaths occur due to gastroenteritis in children <5 years of age [1,2]. Numerous viral agents such as rotavirus, norovirus, astrovirus and adenovirus largely affect children causing gastroenteritis episodes, among which rotavirus and norovirus infections occupy the apex position [3,4,5,6].

Rotavirus (RV), belonging to the family *Reoviridae*, possess 11 double-stranded segments of RNA that encode six structural viral proteins (VP1, VP2, VP3, VP4, VP6, VP7) and five/six non-structural proteins (NSP1–NSP5/6) [7]. Based on the antigenic properties of the major inner capsid protein (VP6), RVs are subdivided into eight well-characterized species (A–H) and two putative species viz. I and J [8,9,10]. Humans and other mammalian species are affected by species A, B, C and H rotaviruses and birds by species D, F and G, and species E has been reported exclusively in pigs [7,8,11,12,13,14,15,16,17]. The newly-proposed species I is reported in dogs [18] and cats [19], whereas species J is found in bats [10].

Rotavirus C (RVC) has emerged as a common enteric viral infection, affecting both humans and animals worldwide [20,21,22]. In contrast to RVA, RVCs have quite different epidemiology, affecting both neonatal and adult human populations [23]. Since the first isolated strain of RVC, Cowden from piglets of the U.S. in 1980 [24] and subsequent isolation of RVC from humans in 1982 [25], RVCs have been confirmed in cow [26], ferrets [27] and dogs [28] as the cause of both sporadic and outbreak disease.

For classification of RV, a binary classification system based on G (VP7 gene) and P (VP4 gene) was in use [7,29]. These days, a genotyping system developed based on nucleotide sequence identity cut-off values of all 11 genome segments, which provides an excellent detection system for re-assortment events and examining evolution of RVs, is in use [30,31,32]. As per the recommendations of RCWG (Rotavirus Classification Working Group), an RV strain is to be designated as Gx-P[x]-Ix-Rx-Cx-Mx-Ax-Nx-Tx-Ex-Hx for the VP7, VP4,VP6, VP1, VP2, VP3, NSP1, NSP2, NSP3, NSP4 and NSP5 genes, respectively, where ‘x’ denotes the genotype for the particular genotype in Arabic numerals [31,33]. Although, like RV-A, no RCWG-approved classification exist for RVC, the genotyping method based on nucleotide sequence identity cut-off values of all 11 genome segments, as has been adopted for RVA, is in use for RVC [32,34]. To date, limited complete genome sequences are available for the RVC strains. Existing reports confirm that the human RVCs fall into a single genotype for most of the gene segments (G4, P [2], I2, R2, C2,A2, N2, T2, E2 and H2) except for the VP3 gene (M2/3) [32,35].

RVC infections are noted more in adults [36,37]. Notably, the epidemiology and prevalence of RVC as an etiologic agent of diarrhea is limited due to the low level of fecal shedding and lack of sensitive diagnostic assays [20]. A few antigen-based immunoassay using RVC-specific antibodies are in use for the detection of RVC infection [36]. Moreover, VP6 gene-specific RT-PCR assays are used for the group-specific detection of RVCs [14,38]. The complete genome sequencing and sequence analysis of RVCs could provide a better approach to investigate the possible evolutionary relationship of viruses obtained from different host species.

The first detection of human RVC (HuRVC) in India based on serology dates back to 1990s [39]. Although, even after two decades, only a few studies have documented the presence of RVC in focal settings, the exact epidemiological blueprint is yet to be determined for India [1,14,37,40]. Here, we describe the screening of the stool samples from patients who either visited or were hospitalized in Haldwani (state of Uttarakhand, India), a city located at the foothills of the Himalayas. Further, analysis of the selected RVC genes was done for their genetic relatedness using bioinformatics tools. The region of sample collection has been chosen due to the absence of any previous report of RVC from this area, and due to its importance as a junction point of the Himalayas and the Indo-Gangetic Plain, it harbors several important trade and commerce hubs for high altitude areas and is a tourist base as well. Recently, we documented from this region a multiple infection of RVA and RVB with picobirnavirus in a child suffering with gastroenteritis [41] and further the presence of porcine RVC in India [14]. The previously discussed report suggests the possibilities of gene re-assortment events through interspecies transmission [42]. Molecular epidemiological studies support the detection of HuRVCs from neighboring countries of India like Bangladesh [43], Russia [44], Malaysia [45], South Korea [46], Thailand, Nepal [47] and Kenya [48], supporting the importance of comprehensive surveillance in the Indian population. As mixed infections of different strain of RVs are a prerequisite for re-assortment events, co-surveillance of animal and human virus strains is vital to gain a better understanding of the relationships between co-circulating viruses.

## 2. Materials and Methods

### 2.1. Collection of Samples

During 2010–2013, 119 fecal samples were screened for RVC from Haldwani (state of Uttarakhand, India), a city known as the gate way of the Himalayan foothills, located at 29°13’0” N 79°31’0” E with an average elevation of 424 m (1391 feet), having a mixed urban and rural culture of various religions with diverse living systems. Hospital-based (Out Patient Department patients/hospitalized, less than one year of age) diarrheic samples were collected with the respective parent’s consent. A 10% (*w/v*) fecal suspension was prepared with phosphate-buffered saline (pH 7.2) followed by clarification of debris by centrifugation at 6797× *g* for 5 min, and the supernatants were used for the viral RNA extraction.

### 2.2. Viral RNA Extraction and cDNA Synthesis

Total RNA from suspended diarrheic stool samples was isolated using Qiazol reagent (Qiagen GmbH, Hilden, Germany) following the manufacturer’s protocol and quantified on a Nanodrop spectrometer [49]. Extracted RNA (500 ng) was used to prepare a pool of first-strand cDNA by random-priming reverse transcription (RT) using recombinant MMLV-RT (Promega Corporation, Madison, WI, USA) and random hexamer (Qiagen GmbH, Hilden, Germany) at 37 °C. Enzymatic activity of the MMLV-RT was stopped by keeping the cDNA reaction mixture at 80 °C for 3 min.

### 2.3. Diagnostic VP6 Gene-Specific RT-PCR Assay

Presence of RVC in diarrheic stool samples was detected by using the RT-PCR assay targeting the VP6 gene with primers RVC-VP6-DF; 5′-ARTCHGTTCTATGYGATTC-3′ [14] and BMJ44; 5′-AGCCACATAG TTCACATTTC-3′ [20] (Supplementary Data 1). The diagnostic primers were expected to amplify a 340-bp amplicon. PCR conditions were optimized as described previously [14] with SapphireAmpFast PCR master mix (Takara Bio Inc., Shiga, Japan). These samples were also subjected to screening for other infectious viral causes of gastroenteritis viz. calicivirus, astrovirus and picobirnaviruses following the primers and procedures outlined in the published reports [50,51,52].

### 2.4. Amplification, Cloning and Sequencing of VP6, VP4, VP7 and NSP4 Genes

**Figure 1:**
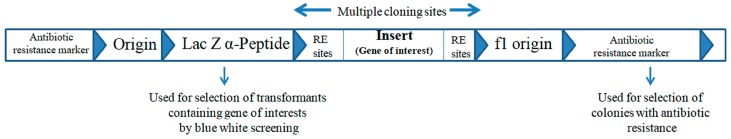
Schematic representation of pDrive cloning vector map (3.85 Kb). The abbreviations refers as RE sites—restriction enzyme sites, f1- origin of replication.

Characterization of the selected RVC strain (HuRVC/H28/2013/India) was done by amplification of the structural capsid genes (VP6, VP4 and VP7) and non-structural enterotoxin gene (NSP4). Primers used for the amplification are mentioned in the Supplementary Data 1. The published PCR conditions for the amplification of individual genes were followed viz. for the VP6 gene [53], VP4 and VP7 genes [43] and NPS4 gene [14]. RT-PCR amplicons were visualized under a UV trans-illuminator after resolving in 1.5% agarose gel electrophoresis. Desired amplified products were cloned into the pDRIVE (Qiagen GmbH, Hilden, Germany, Figure 1) cloning vector and transformed in *Escherichia coli* DH5α competent cells [54]. The recombinant plasmids were obtained using the GeneJET plasmid Miniprep kit (Thermo Fisher Scientific, Waltham, MA, Vilnius, Lithuania). Positive recombinant clones were sequenced by the Bigdye terminator Sanger sequencing method in an ABI 3730 x l sequencer (Eurofins Genomic India Ltd., Bangalore, India). The sequences were deposited in GenBank with Accession Numbers MG553198 (NSP4 gene), MG553199 (VP4 gene) and MG553200 (VP6 gene).

### 2.5. Phylogenetic Analysis and Pair-Wise Sequence Distance for VP6, VP4 and NSP4 Genes

The pair-wise similarity among the nucleotide/amino acid sequences were calculated for the VP6, VP4 and NSP4 genes after aligning the sequences by the Clustal V program in MegAlign software of the DNASTAR software package. For the genetic relatedness study, representative full-length genes (VP6: *n* = 47; NSP4: *n* = 26; VP4: *n* = 30) of human, cattle, pig, dog and ferret RVC strains were retrieved from the NCBI database (Supplementary Data 2) [55]. Phylogenetic analysis was performed using the maximum likelihood method (1000 bootstrap replicates) in MEGA 6 software (v 6.06) [56]. The suitable dendrogram analysis model was identified as described earlier [13] using the find best DNA/protein model tool available in MEGA 6 (v 6.06), confirmed with the FindModel online tool [57].

### 2.6. Determination of N-Glycosylation Sites

The *N*-glycosylation sites on the VP6, VP4 and NSP4 proteins of the HuRVC/H28/2013/India strain were predicted by using a NetNGlyc 1.0 server [58]. These sites were compared with available human RVC sequences (Supplementary Data 2).

## 3. Results and Discussion

### 3.1. Detection of RVC

**Figure 2:**
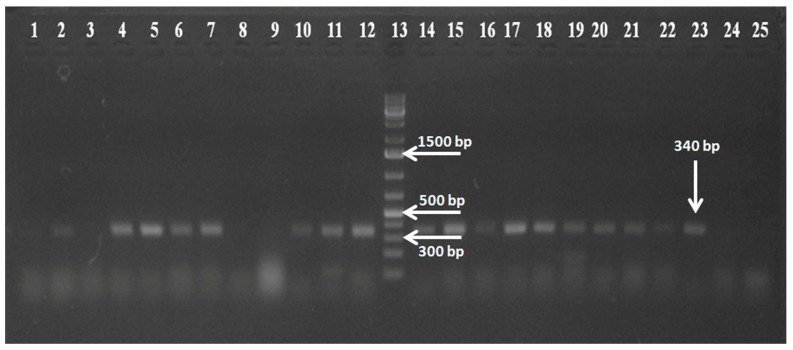
Agarose gel electrophoresis of RT-PCR products showing the 340-bp amplicon confirming the presence of the rotavirus C (RVC). Lanes 1–11 and 14–24: test samples screened for RVC; Lane 12: positive RVC plasmid control; Lane 13: 1-kb plus gene ruler (Thermo Scientific); Lane 25: non-template control.

Among the total 119 samples, 60 were found positive for RVA (unpublished data), and one sample out of 60 was found with picobirnavirus genogroup I (PBV GG-I), RVB and RVA co-infection [41]. Of the, 119 samples, 38 (31.93%) were found positive for RVC in the *VP6* gene-based RT-PCR (Figure 2). Preceding reports from different Asian countries showed both sporadic and outbreak cases with comparatively less prevalence of RVC, which may be to the lack of sensitive detection assay availability [43,44,45,46,47,48]. In the previously mentioned reports from India, serological prevalence of 0.43% and 25.32% was reported by Brown et al. [39] and Mukhopadhya et al. [40], respectively, from southern India (Vellore). The percent molecular detection of RVC by RT-PCR was 8.6% (in the outbreak cases) and 0.7% (sporadic cases) from western India [1] and 3.33% from Delhi [37]. Moreover, the molecular detection of RVCs throughout the world shows a range between 0.3% and 23.7% [1].The present study foresees a high prevalence (31.93%) and supports a diverse epidemiology of RVC in north India among the children of less than one year of age in contrast to the earlier studies, which could emphasize the occurrence of RVC in adults [36]. Notably, none of the sample detected as positive for RVC showed co-infection with screened viruses (calicivirus, astrovirus and picobirnavirus).

### 3.2. Nucleotide and Amino Acid Sequence Analysis

One sample (designated as HuRVC/H28/2013/India) out of the 38 RVC positive samples got amplified partially for the VP6 and VP4 genes and the complete CDS (coding DNA sequence) region for the NSP4 gene using the reported primers (Supplementary Data 1). The absence of intact virus particles or degraded viral RNA (repeated freeze thawing of biological sample or inherent nucleases present in fecal matter) may be a cause for the inability to amplify full-length VP6 and VP4 gene of the virus with otherwise well-established primers. As observed in other studies, cross-priming or primer-binding failures at the 3′ end of the primer may also cause the inability to obtain the full-length genome sequence [59,60].

#### 3.2.1. Structural Gene (VP6)

**Figure 3:**
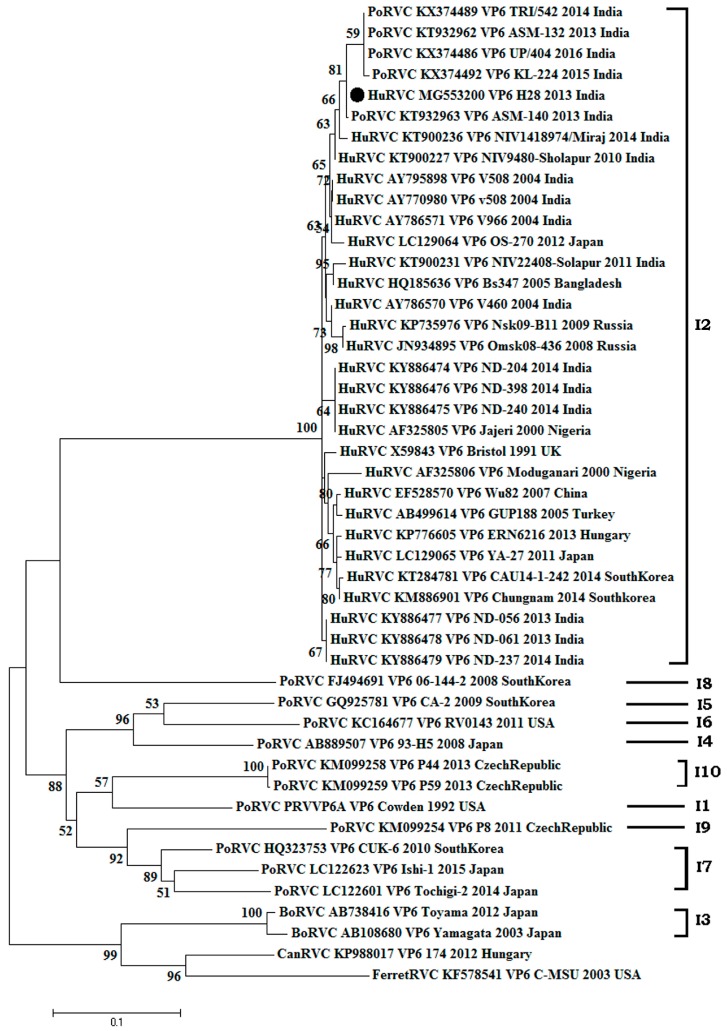
Phylogenetic analysis of rotavirus C based on VP6 genes (831 nucleotide) of human, swine, cattle, dog and ferret origin at the nucleotide level. The Tamura-3+G algorithm was identified using the find best DNA/protein model tool available in MEGA 6, which was confirmed with the FindModel online tool [57]. Numbers on branches indicate the percentages of bootstrap support from 1000 replicates. VP6 gene-based I typing of RVC is denoted along with clusters. Host species depicted are human (Hu); cattle (Bo); pig (Por)); dog (Can); and ferret. Strains/isolates are represented according to their host species, accession number, gene, strain, year of isolation and country of origin. The isolate of the current study is denoted by the solid dot. Genotype designations are shown on the right. The scale bar indicates nucleotide substitutions per site.

The amplified VP6 gene of the HuRVC/H28/2013/India strain has 831 nucleotides (521st–1352nd position), encoding a protein of 228 amino acids in length. We failed to get the sequence stretch from the 5’-end of the VP6 gene. The phylogenetic tree (*n* = 47) of the VP6 gene consisted of two major clades (Figure 3). One contained bovine, ferret and canine RVCs and the other consisted of porcine, human and human-like porcine RVCs. As per the cut-off value established for genotype determination, the HuRVC/H28/2013/India strain clustered with human RVCs (I2) along with all other Indian human RVCs, showing their common ancestry [32]. The study isolate clustered along with Indian human origin (AY795898, AY770980, AY786571, AY786570; Barman and Naik [61]), and KT900227, KT900231, KT900236; [1]) and human-like porcine RVCs (KX374486, KX374489, KX374492, KT932962, KT932963; [14]) in the I2 lineage. Although coming in the same I2 lineage, some of the Indian human RVC sequences reported recently from the Delhi region, Northern India (KY886474-79; [37]) showed comparatively distant phylogenetic relatedness with all other previously-reported Indian RVC sequences. Further, our results corroborated the findings of Tiku and co-workers [37] where an independent evolution of human RVCs of Indian origin at the nucleotide level based on the VP6 gene was noted. The HuRVC/H28/2013/India strain showed the highest sequence similarity with recently-isolated Indian human-like porcine RVC from Assam, PoRVC/ASM140/2013/India (nt: 99.9%, aa: 99.1%) and PoRVC/ASM132/2013/India (nt: 98.7%, aa: 95.7%) (Supplementary Data 3). The presence of a similar VP6 gene in porcine and human RVC strains could suggest that the gene may have been reasserted and could indicate zoonotic or anthroponotic transmission. Interspecies transmission of RVC from pig to children has been reported in Brazil [42,58]. In concordance with a previous Indian study [1], the current study isolate clustered with Indian and Bangladeshi RVC isolates, showing a percent of identity of 97.7–98.5% (nt), and 99.1% (aa), respectively. However, in contrast to another study from India [37] where the isolates were more similar to Nigerian isolates, our isolate (HuRVC/H28/2013/India) showed lower similarity to Nigerian isolates (nt: 95.5–97.1%, aa: 98.7%) (Supplementary Data 3). Sequence identity with other animal species is mentioned in Table 1, where the current study isolate shows high sequence similarity with other human RVCs at the nucleotide and amino acid level (>97%). Of note, the percent similarity was low with animal-origin VP6 sequences (bovine, ferret and canine, <90%), except for porcine, wherein a wide range of similarity was seen (84–99%). This shows close relatedness of the current study’s HuRVC strain to the porcine-origin RVC strain. Further, no significant change in the available amino acid sequence of the VP6 gene from HuRVC/H28/2013/India supports the conserved nature of this species-specific protein.

#### 3.2.2. Structural Gene (VP4)

**Figure 4:**
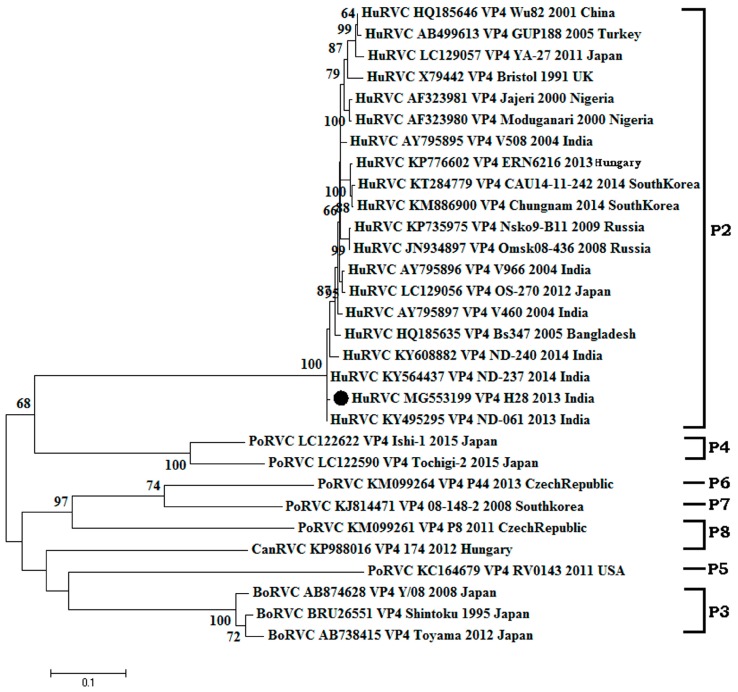
Phylogenetic analysis of rotavirus C based on partial length (1224 bp) VP4 genes of human, swine, cattle and dog origin at the nucleotide level. The Tamura-3+G+I algorithm was identified using the find best DNA/protein model tool available in MEGA 6, which was confirmed with the FindModel online tool [57]. Numbers on branches indicate the percentages of bootstrap support from 1000 replicates. VP6 gene-based I typing of RVC is denoted along with clusters. Host species depicted are: human (Hu); cattle (Bo); pig (Por); dog (Can). Strains/isolates are represented according to their host species, accession number, gene, strain, year of isolation and country of origin. The isolate of the current study is denoted by the solid dot. Genotype designations are shown on the right. The scale bar indicates nucleotide substitutions per site.

**Figure 5:**
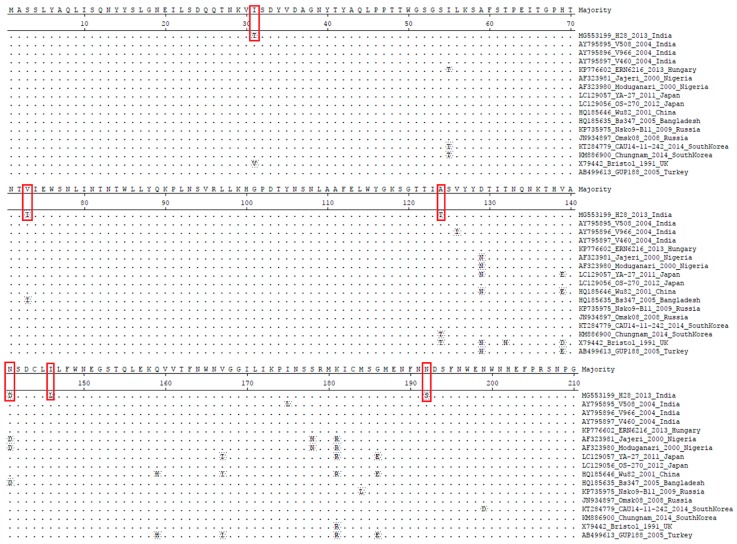
Deduced amino acid sequence alignment of the VP4 genes of the current HuRVC study strain with that of previously-described HuRVC strains. The dots represent the identical amino acids. Six mutations were noticed at positions 31, 73, 124, 141, 146 and 192, indicated in red boxes. Of note, one change was linked with the N-glycosylation site at position 192 (N192S) in the study strain.

The amplified VP4 gene of the HuRVC/H28/2013/India isolate comprised of a 1224-nucleotide (1st–1224th position) long stretch encoded a polypeptide of 408 amino acids. Upon phylogenetic analysis with representative sequences of the RVC VP4 gene (*n* = 30) from different host species, the dendrogram scattered into two major clades, among which one clade contained bovine, canine and porcine RVCs, whereas the another contained all human RVCs (Figure 4). As per the cut-off values stated for genotype determination, the HuRVC/H28/2013/India isolate clustered with human RVCs of the P2 type. Sequence similarity revealed that the study sequence showed the least sequence identity (nt: 94.3%, aa: 95.6%) with the Japanese isolate (LC129057) and highest (nt: 97%, aa: 97.6%) with the Bangladeshi isolate (HQ185635) as determined through the sequence distance in MegAlign software (Supplementary Data 4). Other Indian RVC VP4 strains reported in the study of Tiku et al. [37] were not taken into consideration for analyzing the percentage of identity due to the small nucleotide length. Analyzing the current study strain with the earlier Indian study sequence may lead to misinterpretation of the obtained data. Even though the current study strain shared the same clade as the sequences reported by Tiku and co-workers [37], the isolate of the present study showed a higher sequence identity (96.4–96.9%) (Supplementary Data 4) with other RVC VP4 gene sequences from India, which is not in accordance with the observation by the former report. According to the report, their isolates were more similar to Korean RVCs; however, the isolate HuRVC/H28/2013/India showed more identity to the Bangladeshi and Indian isolates. Though Indian isolates possess a common ancestor, by virtue of the divergence in nucleotide, they have been placed in different sub-clades (Figure 4). Additionally, upon analysis, six point mutations were observed in the amino acid sequence of the current study isolate, i.e., I31T, V73I, A124T, N141D, I146L and N192S, which may have a role in shifting the antigenicity of the circulating RVCs (Figure 5). However, further work is needed to elucidate their position on the protein surface. Sequence identity of the VP4 gene with other animal species is mentioned in Table 1, where the current study strain shows quite high similarity (95.6–97.6%) at the amino acid level with other human RVCs as compared to animal RVCs (60.4–68.7%). This further proves the human origin of our study strain. Further, more divergence in the outer capsid protein VP4 gene in comparison to inner capsid protein VP6 among Indian RVC isolates proves the rapid evolving nature to escape selection pressure.

#### 3.2.3. Non-Structural Gene NSP4

**Figure 6:**
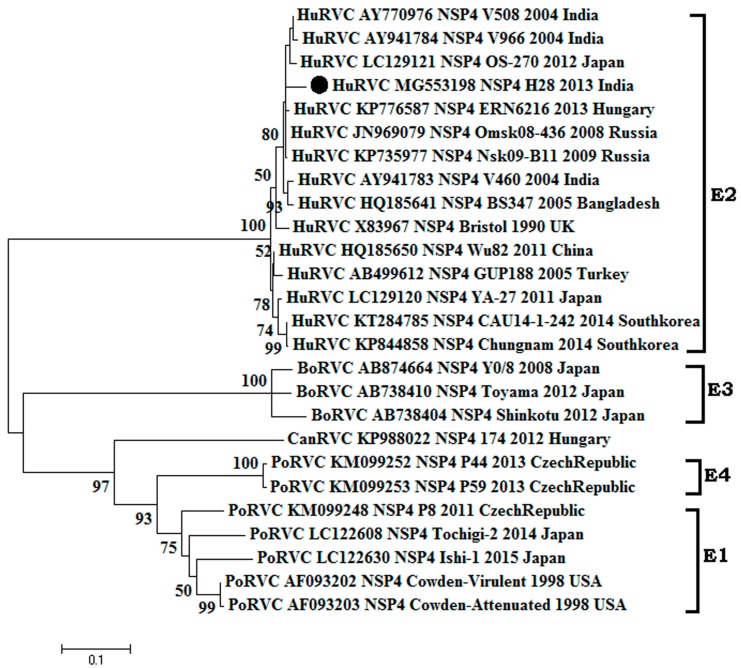
Phylogenetic analysis of species C rotavirus based on NSP4 genes (450 nt) of human, swine and cattle origin at the nucleotide level. The Tamura-3+G+I algorithm was identified using the find best DNA/protein model tool available in MEGA 6, which was confirmed with the FindModel online tool [57]. Numbers on branches indicate the percentages of bootstrap support from 1000 replicates. VP6 gene-based I typing of RVC is denoted along with clusters. Sub-clades within human type RVC are depicted in square brackets. Host species depicted are: human (Hu); cattle (Bo); pig (Por). Strains/isolates are represented according to their host species, accession number, gene, strain, year of isolation and country of origin. The isolate of the current study is denoted by the solid dot. Genotype designations are shown on the right. The scale bar indicates nucleotide substitutions per site.

**Figure 7:**
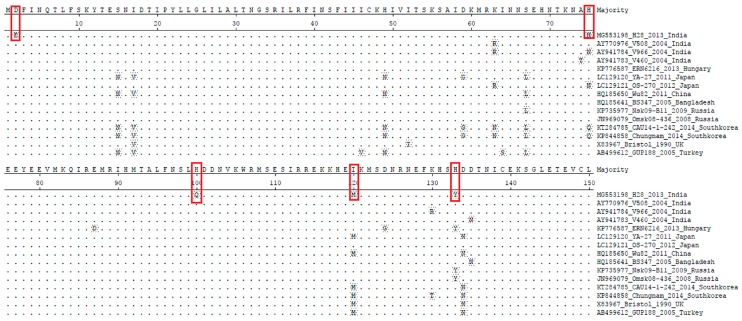
Deduced amino acid sequence alignment of the NSP4 genes of the current study HuRVC strain with that of previously-described HuRVC strains. The dots represent the identical amino acids, and five changes were noticed at positions 2, 75, 100, 120, 133 in the current study’s RVC isolate, indicated in red boxes.

The RVC strains NSP4 gene sequenced in this study has a length of 472 nucleotides, encoding a protein of 150 amino acids in length (complete ORF). The NSP4 gene of human RVC is smaller than that of RVA (175 amino acid) [62] and larger than that of bovine RVC (146 amino acid) [63] The sequence identity at the nucleotide and amino acid level revealed that the study isolate had 91.4–96% and 90.2–95.1% identity, respectively, with the available human RVC sequences. The highest sequence identity (nt: 96%, aa: 95.1%) was observed with Russian isolates (KP735977 and JN969079) and the least identity with Korean isolate Chungnam (nt: 91.4%, aa: 90.2) (Supplementary Data 5). Upon phylogenetic analysis (*n* = 26), all RVC isolates clustered into two major clades, i.e., one which contains human RVCs exclusively and another containing RVC isolated from animals (bovine, porcine and canine) (Figure 6). As per the cut-off value established for the E genotype, HuRVC/H28/2013/India clustered with human RVCs of the E2 genotype. In comparison to the RVC isolates in the animal RVC clade, the isolates in the human RVC clade were more similar. In animal RVC clades, further sub-clades were seen (bovine, canine and porcine subclades). The study strain showed higher genetic relatedness to Eurasian RVC strains (strains from Hungary, Russia, the U.K. and Bangladesh) than to the more geographically-close Chinese RVC strains (Figure 6). The amino acid sequence analysis of the NSP4 gene from HuRVC/H28/2013/India showed point mutations like D2E, H75N, H100Q, I120M and H133Y (Figure 7). Although, the NSP4 gene’s functional role has not been deciphered yet for RVC, the changes at position 120 and 133 might affect the functional property of the NSP4 gene as the amino acid sequence from 114 to 135 is considered responsible for enterotoxin activity and the binding capability of virus with caveolin, which help in the budding of the virus particles in RVA infection [62]. Nevertheless, this has to be proven through site-directed mutagenesis studies. These two point mutations (I120M and H133Y) lie in the well-described VP4 binding domain (described for RVA strains) [64]. This report describes the full-length NSP4 gene from an Indian human RVC. Sequence identity of the VP4 gene with other animal species is mentioned in Table 1, as for the VP4 gene, the NSP4 gene of the current study also shows higher amino acid identity (90.2–95.4%) to other human RVCs as compared to animal RVCs (54.9–62.1%).

#### 3.2.4. Structural Gene VP7

In the current study, we failed to amplify the VP7 gene from any of the RVC-positive samples. A similar observation was noticed by Tiku et al. [37], where they could only amplify 50% of the isolate’s VP7 and VP4 genes. This may be due to the sequence variations observed in the circulating Indian RVC strains; however, newer approaches viz. adaptor ligation amplification could solve this problem.

### 3.3. Prediction of N-Linked Glycosylation Pattern

The prediction and analysis of N-linked glycosylation have gained intense interest in recently due to the association of its relevance with the overall understanding of the virus biology, its ability to impart various advantages to virus survival, folding of the proteins, replication, maturation processes and also virulence. Here, we intended to know the possible glycosylation sites by analyzing the human RVC strains using the NetNGlyc 1.0 Server [58]. In the VP6 protein analysis, we predicted N-glycosylation at positions 36, 64, 76, 89 and 205, and these remained unaltered in the VP6 gene of the current study isolate as well; while in the VP4 protein, there was a deletion of the *N*-glycosylation site (N192S) (Figure 5). Being an outer capsid protein, the predicted deletion in glycosylation (N192S) may alter the virus attachment and entry into the host cell, but needs further studies in this context. The NSP4 in RVs is a heavily glycosylated viral protein [62], and that was also observed during the current analysis of available human RVC NPS4 sequences, where conserved N-glycosylation sites were seen in the NSP4 protein at 5–7 (NQT), 32–35 (NGS) and 65–67 (NNS) [64]. This study provides a preliminary addition to the available knowledge, which in particular is lacking with respect to Indian RVC isolates.

## 4. Conclusions and Future Perspectives

A number of studies on RVC from all over the world have shown growing concern about RVC as one of the causes of severe acute gastroenteritis in human and animals [43,46,65,66]. RVCs have been confirmed in cow [26], ferrets [27] and dogs [28] as the cause of both sporadic and outbreak disease. The prevalence data on RVC in humans from the Indian subcontinent is limited. In this study, we report a high presence (31.93%) of RVC in contrast to the earlier reports, which ascertains RVC as an important diarrheagenic pathogen. The genetic analysis of inner capsid protein VP6 and outer capsid VP4 revealed divergence from earlier Indian human RVC isolates. Overall, the findings of this study establish the presence of RVC in the human population of northern India along with insights about the similarity with porcine RVC (based on the VP6 gene), indicating a potential source and an interspecies transmission event. In view of the emerging importance of RVC infection all around the world, there is a need to conclusively define the classification system for RVC as well. A few of the studies have used the classification criterion adopted for RVA, but need approval of the RCWG before their global acceptance. In the future, it is essential to look into RVC prevalence through extensive surveillance program both in human and animal populations to understand the geographical distribution of RVC infections in India.

## Figures and Tables

**Table 1:** Percent of sequence identities of the current study rotavirus C strain (HuRVC/H28/2013/India) VP6, VP4 and NSP4 genes with that of human, porcine, bovine, canine and ferret origin RVC strains.

	Human Strains		Porcine		Bovine		Canine		Ferret	
Gene	Nucleotide %	Amino Acid %	Nucleotide %	Amino Acid %	Nucleotide %	Amino Acid %	Nucleotide %	Amino Acid %	Nucleotide %	Amino Acid %
VP6	95.5–98.6	98.3–99.1	73.6–99.9	84.3–99.1	78.1–78.5	88.7	64	90	64.3	88.3
VP4	94.3–97	95.6–97.6	59.8–65.1	60.4–65.8	61.7–62.6	67.5–68.7	63.8	68.2	-	-
NSP4	91.4–96	90.2–95.4	58.9–61.3	59.5–62.1	55.2–58.5	54.9–56.9	60.4	59.5	-	-

## Supplementary Materials

The following are available online at http://www.mdpi.com/2076-0817/7/1/23/s1.
