# Supplementary material for: Species C Rotaviruses in Children with Diarrhea in India, 2010–2013: A Potentially Neglected Cause of Acute Gastroenteritis
Source: Pathogens. 2018 Feb 17;7(1):23. doi: 10.3390/pathogens7010023 (PMC5874749; doi:10.3390/pathogens7010023)
Supplement: Supplementary file 1 [file pathogens-07-00023-s001.zip › supplementary/Supplementary data 1 primer details.docx]

**Supplementary Data 1**

Table : Primer details used in present study

| Sl no. | Primer pair | Gene | Sequence  5’ to 3’ | Reference strain  (Position) | Amplicon size | References |
| --- | --- | --- | --- | --- | --- | --- |
| 1 | RVC-VP6-DF | VP6 | ARTCHGTTCTATGYGATTC | JQ513881  (47-65) | 340bp | [14] |
|  | BMJ44 |  | AGCCACATAGTTCACATTTC | JQ513881  (367-386) |  | [20] |
| 2 | VP6 FP | VP6 | GGCTTTAAAAATCTCATTCA | KP342039  (2-23) | 1352 bp | [53] |
|  | VP6 RP |  | AGCCACATAGTTCACATTTC | KP342039  (1334-1353) |  | [53] |
| 3 | RVC VP4 FL FP | VP4 | GGCTTAAAAAGTAGAGATCG | KY062651  (1-20) | 1243bp | [43] |
|  | RVC VP4 FL RP |  | CCAGGATATGATCCTACAGG | KY062651  (1224-1243) |  | [43] |
| 4 | RVC NSP4 CDS FP | NSP4 | CTCTACGAAGCAATGGAGTTCATCAA | KX373864  (27-52) | 477 bp | [14] |
|  | RVC NSP4 CDS RP |  | AGCGCAGAAGATTCATAGACA | KX373864  (483-503) |  | [14] |
| 5 | VP7-20F | VP7 | GCTGTCTGACAAACTGGTC | AF323982  (20-38) | 1043 bp | [43] |
|  | VP7-1062R |  | GCCACATGATCTTGTTTACGC | AF323982 (1042 to 1062) |  | [43] |

There are multiple enteric viral co-infections viz. RVA, RVB, PBV has been detected in the children of Haldwani , India during August,2011 (Malik et al., 2014).
