# Supplementary material for: Species C Rotaviruses in Children with Diarrhea in India, 2010–2013: A Potentially Neglected Cause of Acute Gastroenteritis
Source: Pathogens. 2018 Feb 17;7(1):23. doi: 10.3390/pathogens7010023 (PMC5874749; doi:10.3390/pathogens7010023)
Supplement: Supplementary file 1 [file pathogens-07-00023-s001.zip › supplementary/Supplementary file 1.docx]

**Supplementary Data 1**

**Strains (Name, GenBank Accession no. , Country, Year) taken for phylogenetic analysis in present study:**

| Species | Sl  no | Strain | Gene – Accession no. | | | Country | Year  (Submission/collection) |
| --- | --- | --- | --- | --- | --- | --- | --- |
|  |  |  | VP6 | VP4 | NSP4 |  |  |
| Human | 1 | H28 | MG553200 | MG553199 | MG553198 | India | 2017 |
|  | 2 | V508 | AY795898 | AY795895 | AY770976 | India | 2004 |
|  | 3 | V966 | AY786571 | AY795896 | AY941784 | India | 2004 |
|  | 4 | V460 | AY786570 | AY795897 | AY941783 | India | 2004 |
|  | 5 | v508 | AY770980 | ------ | ------ | India | 2004 |
|  | 6 | ND-056 | KY886477 | ------ | ------ | India | 2013 |
|  | 7 | ND-204 | KY886474 | ------ | ------ | India | 2014 |
|  | 8 | ND-237 | KY886479 | KY564437 | ------ | India | 2014 |
|  | 9 | ND-061 | KY886478 | KY495295 | ------ | India | 2013 |
|  | 10 | ND-398 | KY886476 | ------ | ------ | India | 2014 |
|  | 11 | ND-240 | KY886475 | KY608882 | ------ | India | 2014 |
|  | 12 | NIV9480-  Sholapur | KT900227 | ------ | ------ | India | 2010 |
|  | 13 | NIV1418974-  Miraj | KT900236 | ------ | ------ | India | 2014 |
|  | 14 | NIV22408-Solapur | KT900231 | ------ | ------ | India | 2011 |
|  | 15 | ERN6216 | KP776605 | KP776602 | KP776587 | Hungary | 2013 |
|  | 16 | Jajeri | AF325805 | AF323981 | ------ | Nigeria | 2000 |
|  | 17 | Moduganari | AF325806 | AF323980 | ------ | Nigeria | 2000 |
|  | 18 | YA-27 | LC129065 | LC129057 | LC129120 | Japan | 2011 |
|  | 19 | OS-270 | LC129064 | LC129056 | LC129121 | Japan | 2012 |
|  | 20 | Wu82 | EF528570 | HQ185646 | HQ185650 | China | 2007 |
|  | 21 | Bs347 | HQ185636 | HQ185635 | HQ185641 | Bangladesh | 2005 |
|  | 22 | Nsk09-B11 | KP735976 | KP735975 | KP735977 | Russia | 2009 |
|  | 23 | Omsk08-436 | JN934895 | JN934897 | JN969079 | Russia | 2008 |
|  | 24 | CAU14-1-242 | KT284781 | KT284779 | KT284785 | SouthKorea | 2014 |
|  | 25 | Chungnam | KM886901 | KM886900 | KP844858 | Southkorea | 2014 |
|  | 26 | Bristol | X59843 | X79442 | X83967 | UK | 1991 |
|  | 27 | GUP188 | AB499614 | AB499613 | AB499612 | Turkey | 2005 |
| Porcine | 28 | UP/404 | KX374486 | ------ | ------ | India | 2016 |
|  | 29 | TRI/542 | KX374489 | ------ | ------ | India | 2014 |
|  | 30 | KL-224 | KX374492 | ------ | ------ | India | 2015 |
|  | 31 | ASM-132 | KT932962 | ------ | ------ | India | 2013 |
|  | 32 | ASM-140 | KT932963 | ------ | ------ | India | 2013 |
|  | 33 | Ishi-1 | LC122623 | LC122622 | LC122630 | Japan | 2015 |
|  | 34 | Tochigi-2 | LC122601 | LC122590 | LC122608 | Japan | 2014 |
|  | 35 | CUK-6 | HQ323753 | ------ | ------ | SouthKorea | 2010 |
|  | 36 | 08-148-2 | ------- | KJ814471 |  | SouthKorea | 2008 |
|  | 37 | Cowden | PRVVP6A | ------ | ------ | USA | 1992 |
|  | 38 | Cowden virulent | ------ | ------ | AF093202 | USA | 1998 |
|  | 39 | Cowden avirulent | ------ | ------ | AF093203 | USA | 1998 |
|  | 40 | P8 | KM099254 | KM099261 | KM099248 | CzechRepublic | 2011 |
|  | 41 | 93-H5 | AB889507 | ------ | ------ | Japan | 2008 |
|  | 42 | CA-2 | GQ925781 | ------ | ------ | SouthKorea | 2009 |
|  | 43 | RV0143 | KC164677 | KC164679 | ------ | USA | 2011 |
|  | 44 | 06-144-2 | FJ494691 | ------ | ------ | SouthKorea | 2008 |
|  | 45 | P44 | KM099258 | KM099264 | KM099252 | CzechRepublic | 2013 |
|  | 46 | P59 | KM099259 | ------- | KM099253 | CzechRepublic | 2013 |
| Bovine | 47 | Toyama | AB738416 | AB738415 | AB738410 | Japan | 2012 |
|  | 48 | Yamagata | AB108680 | ----- | ------- | Japan | 2003 |
|  | 49 | Shintoku | ------ | BRU26551 | AB738404 | Japan | 1995, 2012 |
|  | 50 | Y/08 | ------ | AB874628 | AB874664 | Japan | 2008 |
| Canine | 51 | 174 | KP988017 | KP988016 | KP988022 | Hungary | 2012 |
| Ferret | 52 | C-MSU | KF578541 | ------ | ------ | USA | 2003 |
